# Deregulation of miR-100, miR-99a and miR-199b in tissues and plasma coexists with increased expression of mTOR kinase in endometrioid endometrial carcinoma

**DOI:** 10.1186/1471-2407-12-369

**Published:** 2012-08-24

**Authors:** Anna Torres, Kamil Torres, Anna Pesci, Marcello Ceccaroni, Tomasz Paszkowski, Paola Cassandrini, Giuseppe Zamboni, Ryszard Maciejewski

**Affiliations:** 1 Laboratory of Biostructure, Chair and Department of Human Anatomy, Medical University of Lublin, Jaczewskiego 4, Lublin, Poland; 2 III Chair and Department of Gynecology, Medical University of Lublin, Jaczewskiego 8, Lublin, Poland; 3 General and Oncologic Surgery Department, Lublin County Specialist Hospital, Al. Kraśnicka 100, Lublin, Poland; 4 Department of Pathology, Ospedale Sacro Cuore Don Calabria, Via don A. Sempreboni 5, Negrar, Verona, Italy; 5 Gynecologic Oncology Division, International School of Surgical Anatomy, Ospedale Sacro Cuore Don Calabria, Via don A. Sempreboni 5, Negrar, Verona, Italy; 6 Department of Obstetrics & Gynecology, European Gynecology Endoscopy School, Ospedale Sacro Cuore Don Calabria, Via don A. Sempreboni 5, Negrar, Verona, Italy; 7 Department of Oncology, Ospedale Sacro Cuore Don Calabria, Via don A. Sempreboni 5, Negrar, Verona, Italy; 8 Department of Pathology, University of Verona, Piazzale L.A. Scuro 10, 37134, Verona, Italy; 9 II Chair and Department of Surgery, Medical University of Lublin, Staszica 16, 20-081, Lublin, Poland

**Keywords:** mTOR, microRNA, miRNA, Endometrial cancer, Plasma

## Abstract

**Background:**

Alterations of mTOR gene expression have been implicated in the pathogenesis of endometrioid endometrial cancer however only few studies explored the cause of increased mTOR activation in this malignancy. miRNAs are small, noncoding RNAs, which were proven to regulated gene expression at the posttranscriptional level. The study aimed to explore deregulation of miRNAs targeting mTOR kinase (miR-99a, miR-100 and miR-199b) as a possible cause of its altered expression in EEC tissues. In addition expression of the three miRNAs was investigated in plasma of EEC patients and was assessed in terms of diagnostic and prognostic utility.

**Methods:**

We investigated expression of mTOR kinase transcripts in 46 fresh tissue samples. Expression of miR-99a, miR-100 and miR-199b was investigated in the same group of fresh samples, and in additional 58 FFPE sections as well as in 48 plasma samples using qPCR. Relative quantification was performed using experimentally validated endogenous controls.

**Results:**

mTOR kinase expression was increased in EEC tissues and was accompanied by decreased expression of all three miRNAs. Down-regulation of the investigated miRNAs was discovered in plasma of EEC patients and miRNA signatures classified EEC tissues (miR-99a/miR-100/miR-199b) and plasma (miR-99a/miR-199b) samples with higher accuracy in comparison to single miRNAs. We also revealed that miR-100 was an independent prognostic marker of overall survival.

**Conclusions:**

We conclude that increased expression of mTOR kinase coexists with down-regulation of its targeting miRNAs, which could suggest a new mechanism of mTOR pathway alterations in EEC. In addition, our findings implicate that miRNA signatures can be considered promising biomarkers for early detection and prognosis of endometrioid endometrial carcinoma.

## Background

Endometrial cancer is the most common malignant tumor of the female reproductive tract and the fourth most often occurring malignancy in the female population of developed countries [1,2]. The incidence of endometrial cancer has increased in the Great Britain, as it was reported by Evans et al. [3]. Similar trend was also observed in Poland basing on the data provided by Polish Cancer Registry [4]. Most endometrial cancers are diagnosed in early clinical stages and can be managed surgically with good results, however there is still a number of cases with bad prognosis due to lack of effective treatment for advanced, recurrent or disseminated disease. Approximately 80% of endometrial cancers are of endometrioid histology [5]. Etiology of endometrioid endometrial tumors has been connected with alteration of several genes [6,7]. In particular alterations of PTEN (phosphatase and tensin homologue) gene and its product were widely investigated and loss of PTEN activity was proven to induce increased AKT (v-akt murine thymoma viral oncogene homolog; rac-beta serine/threonine protein kinase) and mTOR activity [8-10].

mTOR (mechanistic target of rapamycin) gene is located on chromosome 1 (locus 1p36.2) and encodes for the serine/threonine kinase, which plays a role in cellular response to various stress factors including decreased ATP level, hypoxia and DNA damage. mTOR is involved in the regulation of translation process, cellular proliferation and differentiation [11,12]. It is of particular interest that mTOR kinase, which integrates several cellular pathways activated in the response to changes in ATP and growth factors levels as well as those responsible for cell growth and proliferation, becomes deregulated in many malignant diseases including endometrial cancer [13-16]. It has been proven that it is activated via AKT phosphorylation both directly and indirectly through inactivation of TSC2 (tuberous sclerosis complex 2) [17]. A negative regulation of mTOR activity involves AMPK (5’-AMP-activated protein kinase), which inhibits mTOR actions through phosphorylation and activation of TSC1/TSC2 complex [18,19]. Increased expression and activity of mTOR kinase in endometrial cancer has been connected with the loss of PTEN, LKB1 (Liver Kinase B1) and TSC2 activity as well as increased expression of Phospholipase D1 [20]. Nevertheless, such alterations were not uniformly reported in all EEC cases therefore other mechanisms could be involved in the regulation of mTOR pathway activity including posttranscriptional regulation of mTOR expression, which involves microRNAs (miRNAs). miRNAs are small, non-coding RNA molecules composed of 19 to 25 nucleotides which were proven to regulate gene expression during posttranscriptional stages [21]. miRNAs play important roles in development, cell proliferation and differentiation as well as cell cycle control. Several miRNAs were also associated with the pathogenesis of various malignancies including endometrial cancer [22]. Guo et al. have recently proven that in mammalian cells miRNAs act predominantly through decreasing target mRNA levels [23]. Therefore down-regulation of miRNAs targeting genes involved in carcinogenesis is likely to favor initiation of malignant transformation. Alterations of genes, which are upstream to mTOR and could be responsible for its activation, are not uniformly present in all EEC cases [5]. Therefore in the presented study we aimed to assess mTOR kinase mRNA levels and to explore possible association between altered expression of mTOR kinase transcripts and its targeting miRNAs, miR-99a, miR-100 and miR-199b, which were chosen basing on the three open-source *in silico* algorithms (Target Scan, DIANA microT v. 3.0, miRanda). Expression of the three miRNAs was also investigated in plasma of EEC patients and was assessed in terms of their diagnostic and prognostic utility.

## Methods

### Patients

Altogether one hundred and twenty two patients were included in the study. Patients were hospitalized in gynecological departments of Medical University of Lublin (Poland) and Ospedale Sacro Cuore Don Calabria (Negrar, Italy). Study encompassed 46 tissue samples and 58 formalin-fixed paraffin-embedded specimens as well as 48 blood samples. Study design was revised and approved by Medical University of Lublin Ethical Committee and informed consent was obtained from each study participant. The EEC group consisted of 77 patients: 73 were included in tissue part of the study, whereas material obtained from the remaining four was used only for miRNA expression analysis in plasma. Control group for the tissue part of the study included 31 participants operated on due to benign gynecological pathologies not connected with endometrium and control group for the plasma part of the study included 14 women with no previous history of cancer or endometrial pathology. All patients with EEC were submitted to total hysterectomy and bilateral oophorectomy and were subsequently treated by radiotherapy and/or chemotherapy according to FIGO guidelines. Lymphadenectomy was performed in 44 patients (57%). None of the patients had received neoadiuvant therapy. 2009 revised FIGO classification was used to determine clinical stage of the disease [24]. Table 1 presents detailed characteristics of the patients included in the study. 85.3% of patients with EEC were postmenopausal, whereas most women in the tissue control group were premenopausal (81.6%). The plasma control group comprised of equal numbers of pre- and postmenopausal cases. 

**Table 1:** Clinicopathological characteristics of the patients

**Characteristic**	**Number of EEC patients**	**Number of control patietns**
Average age (years)	62.8 years ± 10.14*	44.78 ± 7.19*
	(95% CI 59.75 – 64.38)	(95% CI 42.51 – 47.05)
FIGO stage		
IA	32	
IB	18	
II	5	
IIIA	2	
IIIB	3	
IIIC1	10	
IIIC2	5	
IVA	1	
IVB	1	
Grade		
1	29	
2	30	
3	18	
Myometrial invasion		
<0.5 of myometrial thickness	36	
≥0.5 of myometrial thickness	41	
Lymph node metastasis		
absent	15	
present	29	
lymphadenectomy not performed	33	
Relapse		
absent	10	
present	24	

* *T*-test, p < 0.001.

### Samples

Fresh tissues were sampled within 15 minutes from excision of the uterus. Tissues were immediately stored in RNAlater (Ambion) and incubated in this solution for 24 hours in 4°C. Tissues were then stored in -80°C until RNA isolation.

FFPE tissues used for the study were fixed with 10% formalin and stored for maximum 10 years. All the original hematotoxylin and eosin (H&E)-stained sections were reviewed by a gynecopathologist (A.P.). EEC specimens were classified, according to the 2002 WHO classification in G1, G2 or G3. Specimens containing at least 70% of cancer cells, were selected and micro dissected.

Samples of the normal endometrium (NE) included both proliferative and secretory phase epithelium.

All blood samples were collected from antecubital vein using closed blood collection system (S-Monovette (EDTA), Sarstedt) and centrifuged for 15 minutes (3200 rpm, 19°C). Plasma was collected, aliquoted and stored in –80°C.

### RNA isolation

40 to 80 mg of macro dissected tissues stored in RNAlater was homogenized in a rotor -stator homogenizer and RNA was isolated using mirVANA^TM^ miRNA Isolation Kit (Ambion) according to manufacturer’s protocol.

RNA isolation from FFPE tissues was performed using Recover All™ Total Nucleic Acid Isolation Kit for FFPE Tissues (Ambion) according to the protocol provided by the manufacturer. As suggested in the protocol, 10 μm FFPE sections were used for isolation procedure. RNA isolated from tissues underwent DNase treatment using Turbo DNAase Kit (Ambion).

Isolation of RNA from plasma was performed using mirVANA^TM^ PARIS Kit (Ambion) and 400 mL of plasma. 5 fmol/μl of each of the following synthetic *Caenorhabditis elegans* oligonucleotides : cel-miR-39, cel-miR-54, cel-miR-238 was spiked into plasma sample after addition of 2xDenaturating Solution. From that point isolation was performed according to manufacturer’s protocol. Elution was performed with 52.5 μl of RNase, DNase-free water. Isolation of RNA from plasma was conducted in duplicates. RNA was stored in –80°C.

Concentration and purity of RNA was inspected using spectrophotometer (Biophotometer with Hellma Tray Cell, Eppendorf) and RNA integrity was checked using Bioanalyzer 2100 (Agilent Technologies Inc.) and Agilent RNA Nano kit. In case of RNA isolated from fresh frozen tissues only samples with RIN ≥ 6 were used in downstream applications. In case of RNA extracted from FFPE the average RIN was 3.45. 260/280 ratio of all tissue samples ranged between 1.8 - 2.2.

### Reverse transcription of total RNA

Total RNA (500 ng) was reverse transcribed in triplicates using Precision nanoScript Reverse Transcription kit (Primer Design) according to manufacturer protocol. Briefly, a mix of 9 μl of RNA o 1 μl of random nonamers was incubated in 65°C for 5 min. Then the mixture was combined with 2 μl nanoScipt buffer 10x, 1 μl 10 mM nucleotides, 2 μl 100 mM DTT, 1 μl reverse trancriptase and 4 μl water. Reactions were then incubated in 25°C for 5 min, in 55°C for 20 min and in 75°C for 15 min.

Reactions were carried in Master cycler ep gradient S (Eppendorf) and stored in –20°C.

### Real-time qPCR for mTOR and PTEN expression

Amplification of mTOR, PTEN and three reference genes (SDHA, UBC, CYC1) was performed in 20 μL reactions. Each reaction consisted of: 1 μL 20x TaqMan® Gene Expression Assay (mTOR Hs00234508, PTEN Hs02621230_s1, SDHA Hs00188166_m1, UBC Hs00824723_m1, CYC1 Hs00357717_m1, Applied Bios stems), 4 μL cDNA (50 ng), 10 μL 2x TaqMan® Gene Expression Master Mix (Applied Bios stems) and 5 μL water. Reactions were carried out in duplicates in Rotor Gene 6000 2-plex HRM thermo cycler (Corbett Research) using the following protocol: 95°C for 10 min and 45 cycles of 95°C for 15 sec/55°C for 60 sec.

Cq values were determined with the threshold value of 0.03 and automatically defined baseline.

### miRNA Reverse transcription and real-time qPCR

RNA isolated from tissues was reverse transcribed in 7.5 μl reactions, which consisted of 3.5 μL RT Master Mix (0.75 μL 10 x RT Buffer, 0.075 μL 100 mM dNTPs, 0.5 μL Multiscribe^TM^ Reverse Transcriptase, 0.095 μL RNase Inhibitor (20U/μL), 2.08 μL nuclease-free water, 1.5 μL of specific starters and 2.5 μL RNA (5 ng). Plasma RNA was reverse transcribed in 5 μl reactions: 0.5 μL 10 x RT Buffer, 0.05 μL 100 mM dNTPs, 0.33 μL Multiscribe^TM^ Reverse Transcriptase, 0.063 μL RNase Inhibitor (20U/μL), 1.387 μL nuclease-free water, 1 μL specific starters and 1.67 μL of RNA eluate. RT reactions were carried out in triplicates using following protocol: 16°C for 30 min, followed by 42°C for 30 min and 85°C for 5 min in Master cycler ep gradient S (Eppendorf) and stored in –20°C.

qPCR reactions for miRNA expression analysis were prepared as follows: 0.5 μL 20x TaqMan® MicroRNA Assay (assay IDs, miR-99a 000435, miR-100 000437, miR-199b 000500, Applied Bios stems), 4.5 μL RT product (dilution 1:15 for tissues and 1:7.5 for plasma), 5 μL 2x TaqMan® Universal PCR Master Mix (Applied Biosystems).

All qPCR reactions were performed in duplicates in ViiA7 Real–Time PCR System (Applied Biosystems) using the following PCR protocol: 95°C for 10 min and 45 cycles of 95°C, 15 s/60°C, 60 s. Positive and negative control reactions as well as inter–plate calibrator (IPC) reactions were carried out on each plate.

Cq values were determined with the threshold value of 0.2 and automatically defined baseline.

### Normalization of qPCR data

Before statistical analysis qPCR data were normalized with inter-plate calibrators to adjust for run-to-run differences and corrected for reaction efficiency [25].

Efficiency of each primer/probe sets was determined by performing standard curve with six-fold dilution of a pooled cDNA template.

Expression of mTOR and PTEN was then further normalized using geometric mean of the expression of three stable reference genes: SDHA, UBC and CYC1, which were experimentally chosen from the panel of 12 candidate genes. Similarly, tissue miRNA qPCR data were normalized using geometric mean of the expression of three experimentally chosen endogenous controls RNU48, RNU44 and U75. Choice of those specific snRNA was performed from the panel consisting of 12 non-coding RNAs, which were previously described to be stably expressed in tissues or were used in endometrial cancer qPCR studies [26-29].

Additional normalization step was implemented in case of plasma miRNA expression data in order to adjust for the variation in RNA extraction. Raw data were initially normalized to expression of three synthetic oligonucleotides matching the sequences of *Caenorhabditis elegans* miRNAs: cel-miR-39, cel-miR-54, cel-miR-238 using median normalization procedure as it was previously described [30,31].

Five experimentally chosen stable endogenous controls: miR-93, miR-26b, miR-192, miR-103a, miR-142-3p were then used for normalization of plasma miRNA expression data. Reference miRNAs were chosen from the panel comprising nine candidate miRNAs (miR-93, miR-26b, miR-192, miR-103a, miR-142-3p, miR-92a, miR-638, miR-16 and miR-451), which were suggested to be highly and stably expressed in plasma [32-35]. Endogenous controls used for both mRNA and miRNA relative expression studies were chosen using two statistical tools Norm Finder and geNorm and validation studies were performed in groups consisting of 45 tissue samples and 48 plasma samples [36,37].

### Statistical analysis of qPCR data

After normalization data were log transformed. Results are presented as mean with 95% confidence intervals (95% CI) or a fold change (FC) with 95% CI.

D’Agostino-Pearson test and Fisher test were used to assess normal distribution of data and equality of variances, respectively. Comparisons between the groups were performed with Student *t*-test (with or without Welch correction) and ANOVA or with nonparametric tests (Mann–Whitney and Kruskal-Wallis) depending on the results of the normality assessment. Spearman coefficient was applied to determine correlation between expressions of miRNAs.

Receiver-operating characteristics (ROC) curves were constructed and sensitivity, specificity as well as positive and negative predictive values were determined to assess accuracy of miRNA expression in classifying EEC and control samples.

Multivariate logistic regression analysis was applied to inspect if miRNA signatures composed of more than one miRNA present better accuracy in discriminating between tumor and control samples. *χ*^2^ Wald test and Hosmer-Lemeshow test were used to evaluate regression models.

Survival analysis was performed using univariate and multivariate Cox proportional hazard models and Kaplan-Meier estimator. Survival curves were compared with log-rank test. Level of significance was set for p ≤ 0.05 and all statistical tests were two-sided. GenEx 5.3.4. (MultiD) and MedCalc (MedCalc Software) version 12.2.1. were utilized to perform data analysis.

## Results

### mTOR kinase and PTEN mRNA expression in EEC tissues

**Figure 1:**
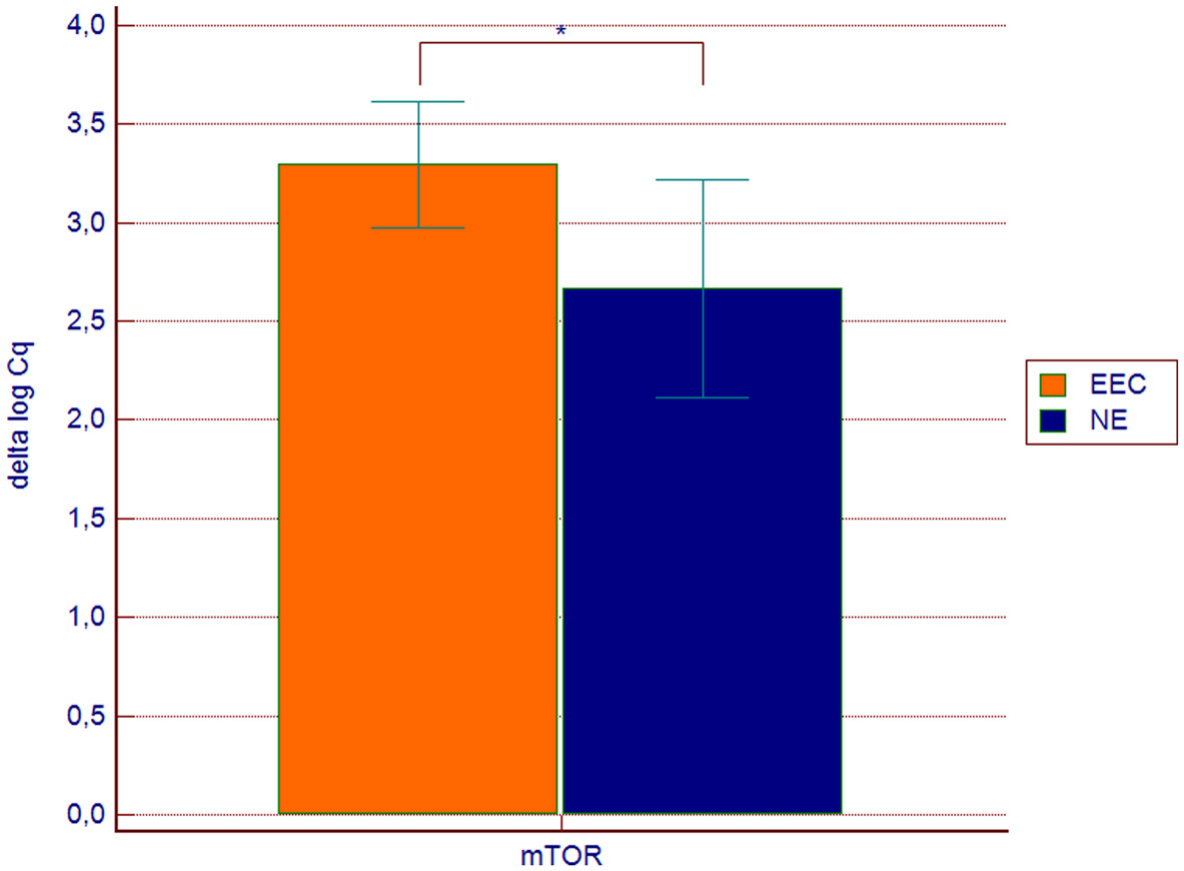
**mTOR gene expression in endometrioid endometrial cancer samples (n = 31) and in the control group (n = 15).** Values are presented in the log scale; * p = 0.033

We found an increased expression of mTOR kinase mRNA in EEC tissues (FC 1.54, 95% CI 1.01-2.36, p = 0.033) (Figure 1). Comparison of groups distinguished based on histological grade, FIGO stage and myometrial invasion did not revealed any significant differences. Nevertheless, when compared separately, G1 samples were not significantly different in comparison to control samples in regards to mTOR expression, whereas in G2 samples or combination of G2 and G3 samples mTOR kinase transcripts levels were significantly up-regulated (p = 0.05 and 0.022, respectively).

Expression of mTOR was also compared between proliferative and secretory endometrium within the control group and no significant differences were found between the groups.

Expression of PTEN mRNA, a negative upstream regulator of mTOR pathway, was not changed in the investigated set of EEC tissues (FC 1.38, 95%CI 0.600–3.040, p = 0.38)

### miRNAs targeting mTOR kinase are decreased in EEC tissues

**Figure 2:**
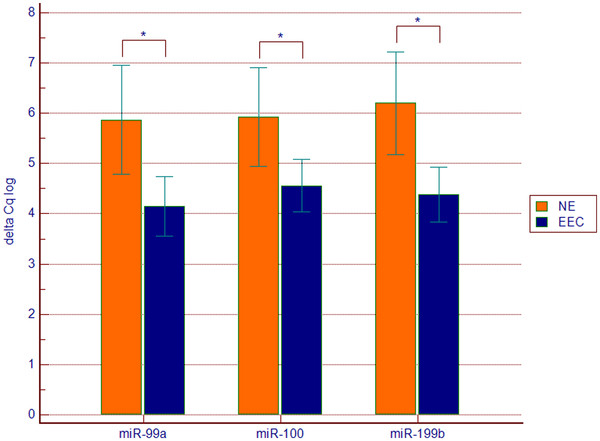
**Expression of miR-99a, miR-100 and miR-199b in endometrioid endometrial cancer (EEC n = 73) tissue samples in comparison to control group (NE n = 31).** Values are presented in the log scale; * p = 0.007 (miR-99a), * p = 0.02 (miR-100), * p = 0.002 (miR-199b)

miR-99a, miR-100 and miR-199b were significantly down-regulated in EEC tissues (Figure 2). The lowest expression was attributed to miR-199b (FC 3.52, 95% CI 1.59-7.79, p = 0.002). In addition, its down-regulation in EEC tissues correlated with advanced histological grade and was the lowest in G3 samples. The difference between G2 and G3 samples was significant (p = 0.04) and the difference between combined G1 and G2 samples and G3 was close to statistical significance (p = 0.056).

miR-99a was more than threefold decreased (FC 3.29, 95% CI 1.49-7.26, p = 0.007) in EEC tissues. Comparison between groups distinguished based on histological grading revealed the lowest miR-99a expression in G3 samples, which was significantly different from G2 samples (p = 0.047) and combined G1 and G2 samples (p = 0.029). Neither miR-99a nor miR-199b expression correlated with other clinic pathological characteristics including FIGO stage, myometrial invasion, lymph node metastasis and relapse.

miR-100 was decreased by the factor 2.56 (9% CI 1.27-5.23, p = 0.02) and no correlation was found between its expression and clinic pathological characteristics of the disease.

Decreased expression of three studied miRNAs significantly correlated with increased mTOR expression in EEC tissues (r = -0.36, p = 0.022 for miR-99, r = -0.36, p = 0.023 for miR-100, r = -0.2, p = 0.023 for miR-199b). In addition there was a significant and very strong correlation between miR-99a and miR-100 expression, both in EEC and control samples groups (r = 0.94, p < 0.0001), whereas no correlation was found between any of the two miRNAs and miR-199b.

Comparison of miR-99a, miR-100 and miR-199b expression performed between proliferative and secretory endometrium within the control group did not reveal any significant differences.

### miRNAs targeting mTOR kinase are up-regulated in plasma of EEC patients

**Figure 3:**
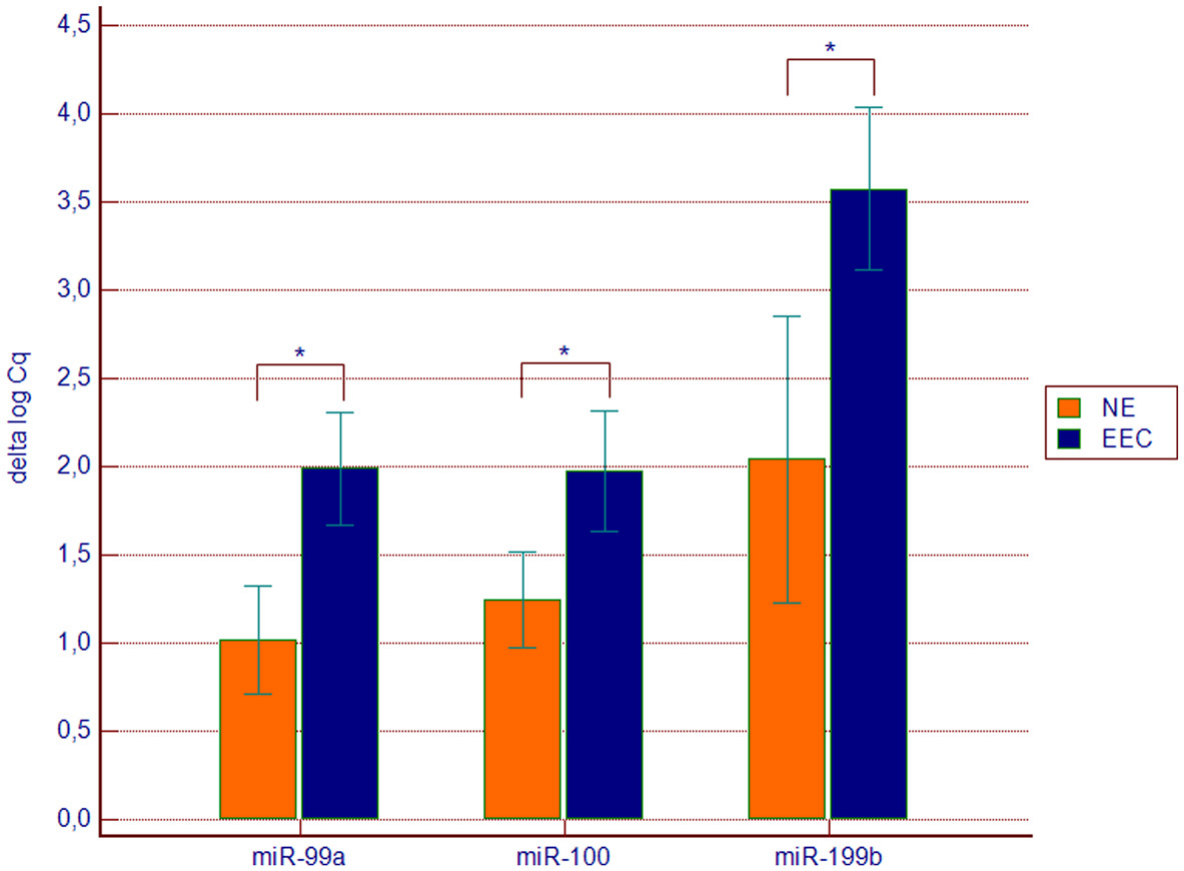
**Expression of miR-99a, miR-100 and miR-199b in endometrioid endometrial cancer (EEC n = 34) plasma samples in comparison to control group (NE n = 14).** Values are presented in the log scale; * p < 0.0001 (miR-99a), * p = 0.001 (miR-100), * p = 0.0007 (miR-199b)

Expression of all three studied miRNAs was significantly increased in plasma of EEC patients (Figure 3). The highest fold change in expression was attributed to miR-199b, which was almost three times increased (FC 2.89, 95% 1.6-5.23, p = 0.0007). Nearly two-fold increase was detected in case of miR-99a expression (FC 1.96, 95% CI 1.37-2.82, p < 0.0001) and miR-100 was 1.65-fold up-regulated (95% CI 1.13-2.42, p = 0.001).

**Figure 4:**
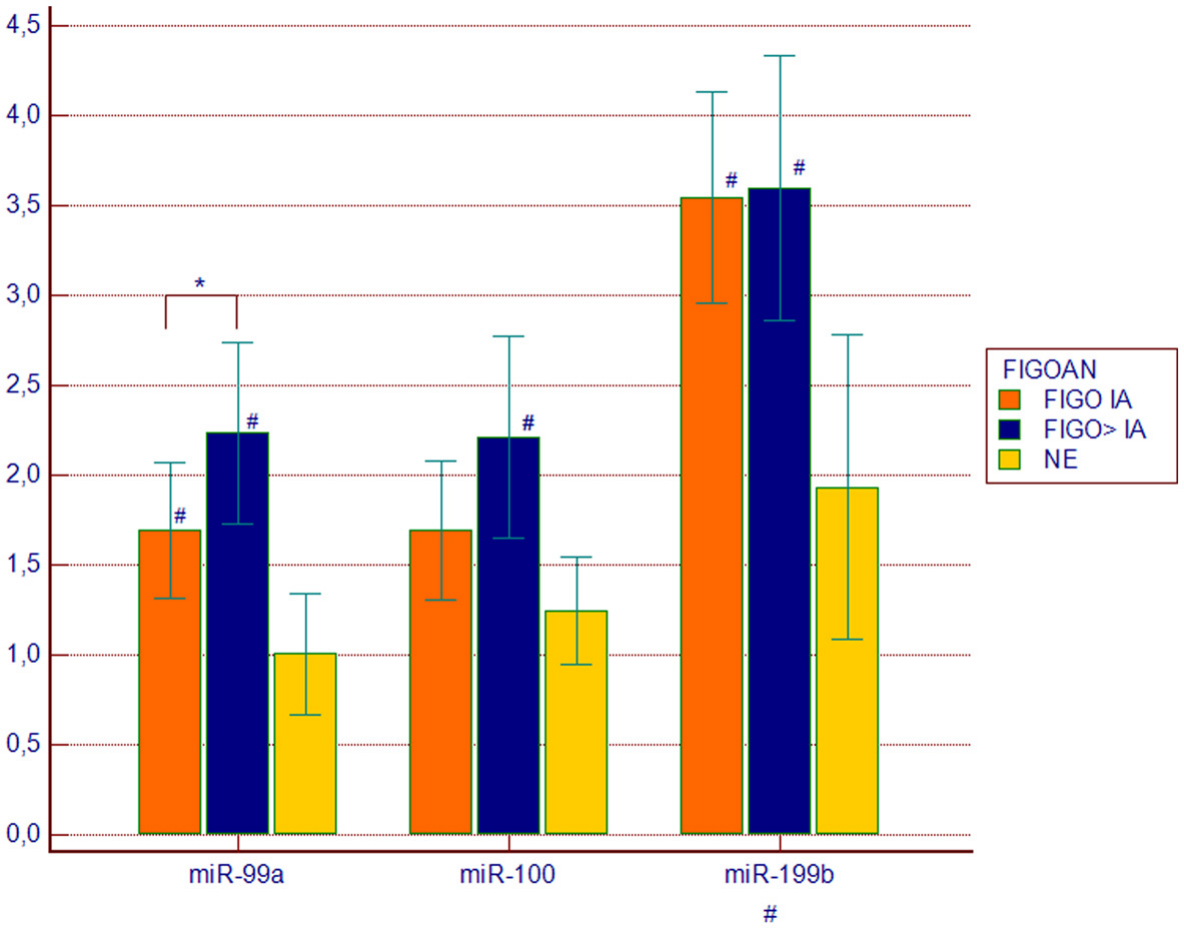
**Expression of miR-99a, miR-100 and miR-199b in endometrioid endometrial cancer (EEC) plasma samples in groups distinguished based on FIGO stage.** FIGOIA (n = 17), FIGO > IA (n = 17), NE, normal endometrium (n = 14). Values are presented in the log scale; * p = 0.039, # p < 0.05

Comparison of groups distinguished based on FIGO stage (FIGO IA vs. FIGO > IA) revealed significantly higher expression of miR-99a in plasma of patients with more advanced disease (p = 0.039, Figure 4). There was no significant association between plasma expression levels of the three miRNAs and grade as well as myometrial invasion.

### Diagnostic and prognostic significance of miR-99a, miR-100 and miR-199b

**Figure 5:**
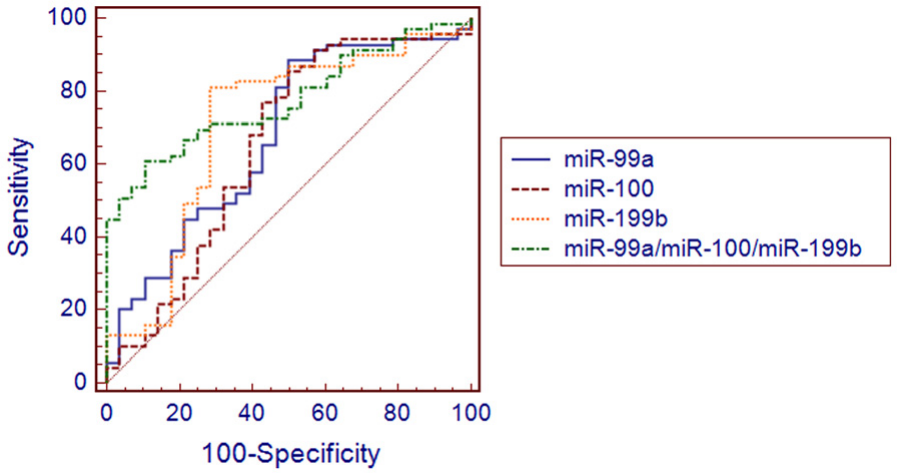
**Receiver operating characteristics curve (ROC) analysis using tissue miRNAs expression for discriminating endometrioid endometrial cancer (EEC) samples (EEC (n = 73) vs. NE (n = 31).** A miRNA signature consisting of three miRNAs yielded area under curve (AUC), which was higher in comparison to single miRNAs (0.774, 95% CI 0.678–0.853, p < 0.0001)

**Figure 6:**
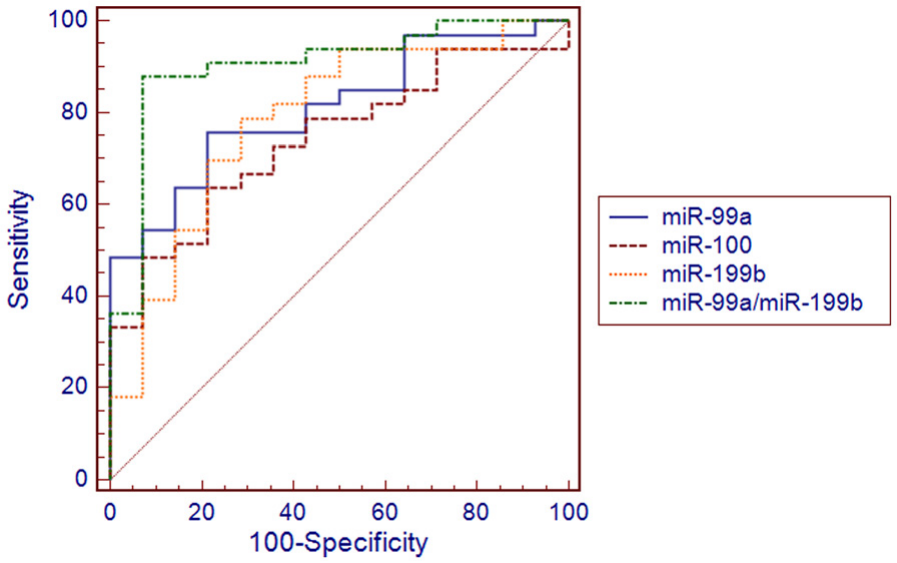
**Receiver operating characteristics curve (ROC) analysis using plasma miRNAs expression for discriminating endometrioid endometrial cancer (EEC) samples.** A miRNA signature consisting of two miRNAs yielded area under curve (AUC), which was higher in comparison to single miRNAs (0.903, 95% CI 0.780–0.970, p < 0.0001, EEC (n = 34) vs. NE (n = 14)

Receiver operating characteristic (ROC) curves were constructed based on miR-99a, miR-100 and miR-199b expression in tissues and plasma and were used to access utility of single miRNAs to differentiate between EEC and control samples. Resulting ROC curves parameters including AUCs, sensitivity and specificity values and positive and negative predictive values were presented in Table 2. Expression of miR-199b yielded the highest area under curve (AUC) value of 0.704 (95% CI 0.606-0.794, p = 0.001) in classifying EEC and normal endometrial tissues (Figure 5). The highest AUC value for detection of EEC plasma samples was attributed to miR-99a (0.810, 95% CI 0.669–0.909, p < 0.0001) (Figure 6). Next, we applied logistic regression analysis in order to assess, if signatures composed of more than one miRNA distinguished EEC and control samples with higher accuracy. The values predicted by obtained models belonged to <0, 1 > interval and represented the probability of EEC. In the applied regression model probability values > 0.5 classified samples as malignant. The model is as follows: *p* = 1/1 + *e*^*-z*^, where *e* is a base of natural logarithm equal to 2.71828…, and *z* is a linear combination of miRNAs expression values (*x*_i_) weighted by the regression coefficients (*b*_i_) derived from the multivariate regression analysis: *z* = *b*_0_ + *b*_1_*x*_1_ + *b*_2_*x*_2_ + … + *b*_n_*x*_n_[38]. A backward stepwise method revealed that tissue expressions of all three miRNAs were independently associated with EEC and had significant influence on the constructed model (p < 0.05). The 3-miRNA signature demonstrated higher sensitivity and specificity in distinguishing between cancer and control tissues samples ( Additional file 1 and Additional file 2) in comparison to single miRNAs. Similarly, backward logistic regression method revealed that plasma expressions of two miRNAs had significant influence on the model constructed to distinguish EEC and control plasma samples. The 2-miRNA signature based on miR-99a and miR-199b expression in plasma was more accurate in comparison to single miRNAs, with 88% sensitivity and 93% specificity (Table 2, Additional file 3 and Additional file 2). 

**Table 2:** Receiver operating characteristics curve (ROC) analysis using tissue and plasma miRNAs expression for discriminating endometrioid endometrial cancer (EEC) samples

**miRNA/miRNA signature**	**AUC [95% CI]**	**p**	**Sensitivity**	**Specificity**	**C**	**+PV**	**-PV**
Tissues (EEC n = 73, NE n= 31)							
miR-99a	0.675 [0.572-0.767]	0.006	0.88	0.5	6.6	81.3	63.6
miR-100	0.652 [0.548-0.746]	0.03	0.86	0.5	6.5	80.8	58.3
miR-199b	0.706 [0.606-0.794]	0.001	0.81	0.71	5.3	87.5	60.6
miR-99a/miR-100/miR-199b	0.774 [0.678-0.853]	<0.0001	0.61	0.89	0.76	93.3	48.1
Plasma (EEC n = 34, NE n = 14)							
miR-99a	0.810 [0.669-0.909]	<0.0001	0.76	0.79	1.23	89.3	57.9
miR-100	0.740 [0.592-0.857]	0.001	0.64	0.79	1.5	33.3	92.8
miR-199b	0.786 [0.642-0.892]	0.0002	0.79	0.71	2.48	86.7	58.8
miR-99a/miR-199b	0.903 [0.780-0.970]	<0.0001	0.88	0.93	0.73	96.7	76.5

*AUC* - area under curve; 95% *CI* – 95% confidence interval; *C* - factor; +*PV* - positive predictive value; -*PV* - negative predictive value.

Univariate Cox regression analysis revealed association between miR-99a (p = 0.034) and miR-100 (p = 0.05) expression in tissues and overall survival. Moreover, miR-99a (p = 0.002) and miR-100 (p = 0.005) were the only independent prognostic markers of overall survival in a multivariate analysis, which included FIGO stage, histological grade, myometrial invasion and the presence of lymph node metastases (Table 3).

**Table 3:** **Univariate and multivariate analyses of overall survivalpredictors for patients with EEC**

	**Univariate analysis**			**Multivariate analysis**		
	**Exp(b)**	**95% CI**	**p**	**Exp(b)**	**95% CI**	**p**
FIGO*	2.583	1.352–4.937	0.004	2.938	0.419 –20.579	0.280
Grade**	1.358	0.685–2.689	0.383	0.526	0.109–2.539	0.426
Nodes	3.129	1.393–7.024	0.006	4.368	0.835–22.849	0.082
Myometrial invasion	0.536	0.272–1.054	0.072	1.454	0.394–5.369	0.576
miR-99a	0.806	0.656–0.989	0.034	0.283	0.129–0.622	0.002
miR-100	0.813	0.657–1.007	0.05	2.940	1.386–6.234	0.005
miR-199b	0.807	0.644–1.012	0.065	0.755	0.485–1.175	0.216

*FIGO - I vs. II-IV; ** G1-G2 vs. G3; Exp(b) hazard coefficient, 95% CI – 95% confidence interval.

**Figure 7:**
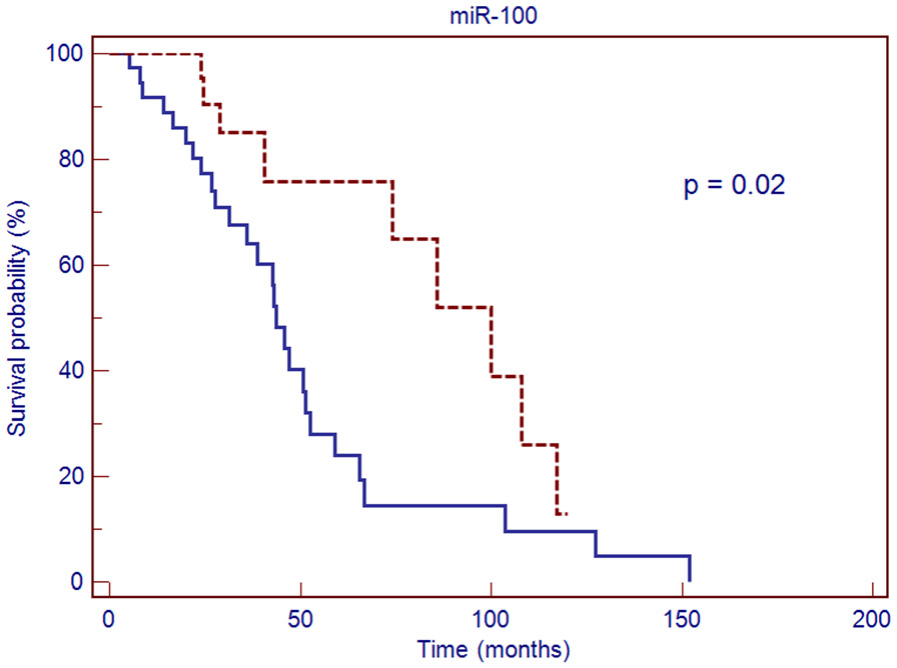
**Kaplan-Meier overall survival curve for patients with endometrioid endometrial cancer (EEC) based on miR-100 expression.** Red line – values above median, blue line – values below median; HR 0.43, 95% CI 0.222–0.830, p = 0.02

In order to generate Kaplan-Meier curves log transformed normalized Cq values were converted into discrete variables by splitting the samples into “high” and “low” expression group using quartile method. Kaplan-Meier estimation and log rank test confirmed the association with the overall survival in case of miR-100 (p = 0.02, HR 0.43, 95% CI 0.222-0.830) and revealed shorter survival times in a group presenting down-regulation of miR-100 expression (Figure 7). In case of miR-99a similar association was observed, though it did not reach statistical significance (p = 0.066, HR 0.497, 95% CI 0.252-0.980).

## Discussion

Several studies reported increased expression of mTOR protein and its phosphorylated form (p-mTOR) in endometrial cancer tissues and cell lines. Study by Darb-Eshafani et al. was one of the first to describe activation of mTOR pathway in a set of human endometrial cancer tissues [16]. Authors found that the activation occurred predominantly in high-grade, high-stage tumors [16]. Similar observation was made by Lu et al., who observed mTOR pathway activation in primary tumors and endometrial cancer cell lines, which was associated with decreased expression of mTOR upstream regulators like TSC2, LKB1 and PTEN [39]. In line with those studies Li et al. reported on activation of mTOR pathway in HEC-1A and Ishikawa cell lines, and found increased expression of mTOR mRNA and higher expression of its downstream substrates in PTEN-deficient Ishikawa cell line [40]. Yoshida et al. pointed at nuclear localization of p-mTOR as an important indicator of poor prognosis and tumor progression, whereas most other authors reported cytoplasmic localization of that kinase [40-42].

A number of studies linked mTOR pathway activation with loss of PTEN and increased AKT expression [39,42]. However Mori et al. did not observe any correlation between expression of phosphorylated AKT and PTEN mutations and expression of its downstream effectors including p-mTOR [43]. Moreover, in the study performed by Yoshida et al. there was no association between activated AKT and p-mTOR expression along with no correlation between AKT expression and cancer progression or outcome [41]. In accordance with those findings Darb-Esfahani et al. observed activation of mTOR in PTEN willed-type cells, in the absence of activated AKT [16].

Basing on the literature indicating mTOR pathway activation in endometrial tumors several mTOR inhibitors have been studied in this cancer [44,45]. Interestingly in the recent, phase II study of temsirolimus in management of patients with recurrent or metastatic endometrial cancer mTOR inhibition did not correlate with PTEN status. In addition authors did not found any relation between expression of phosphorylated AKT, p-mTOR, or phosphorylated S6 and response to treatment or disease progression [46].

Taken together such observations suggest that alternative mechanisms, not related to PTEN/AKT alterations, could play a role in mTOR pathway activation. Based on that assumption we hypothesized that increased expression of mTOR kinase could be caused by alterations in posttranscriptional phases of its biogenesis, which might occur due to disturbed expression of its targeting miRNAs.

Using a quantitive PCR and three experimentally validated endogenous controls we found that mTOR transcripts level was elevated in EEC tissues. In addition we did not observe significant alteration of PTEN mRNA expression, which is a negative upstream regulator of mTOR kinase pathway. Such observation is in accordance with the previous study reporting increased expression of mTOR transcripts and protein in endometrial cancer cell lines as well as with findings presented by Yoshida et al. and Mori et al. [40,41,43]. In addition we have also observed that mTOR mRNA expression tended to be higher in histologically more advanced tumors (G2 and G3 of histological grading). Such finding is in line with the results presented by Darb-Eshafani et al., who observed that mTOR pathway activation was more prevalent in high grade, high stage tumors [16]. Yoshida et al. reported on similar observation, which indicated more pronounced p-mTOR expression in endometrial cancer and correlation between nuclear expression of phosphorylated mTOR and worse prognosis [41]. Contrary to those findings, Choi et al. described significantly decreased p-mTOR expression in deep infiltrating endometrial tumors and indicated that expression of p-mTOR predicted better disease-free survival in endometrial cancer patients [47]. No et al. reported on infrequent mTOR expression (7.1%) in endometrial cancer and did not reveal any correlation between mTOR expression and histological type, grade, stage, myometrial invasion, lymph node metastasis and survival in a large group of 141 patients [48]. In opposition to those observations Wahl et al. observed cytoplasmic p-mTOR expression in 89.2% of type 1 endometrial carcinomas and Mori et al. found moderate and strong expression of phosphorylated mTOR in 81% of endometrioid endometrial cancer samples [42,43]. Such discrepant findings presented by different authors are difficult to interpret. To some extent they could be explained by histological heterogeneity of sample populations used in some of those studies [48]. However, relations between mTOR kinase expression and EEC biology warrant further evaluation and are particularly important in the context of only moderate responses observed in endometrial cancer patients treated with mTOR pathway inhibitors.

In our study increased level of mTOR mRNA was accompanied by down-regulation of miR-99a, miR-100 and miR-199b, which were found to target this gene in the analysis performed using three *in silico* algorithms. No previous study reported on the connection between expression of those miRNAs and mTOR transcripts level in endometrial cancer. However, miR-100 was found strongly down-regulated in clear cell ovarian carcinoma and its altered expression was associated with increased expression of mTOR mRNA and protein. Moreover, a direct link between miR-100 and mTOR expression was proven experimentally in the same study. The authors also found that the increase of intracellular miR-100 level enhanced therapeutic effect of mTOR inhibitor in clear cell ovarian carcinoma cell line [49]. Li et al. described down-regulation of miR-99a in hepatocellular carcinoma, which was accompanied by increased expression of its two target genes mTOR and IGF1R. The authors demonstrated, that transfection with miR-99a resulted in mTOR pathway inhibition and cell cycle arrest in that cancer [50]. Chen at al. obtained similar results and observed down-regulation of both mTOR and IGF1R after transfection with miR-99 family members in head and neck squamous cell carcinoma [51]. A connection between decreased expressions of miR-99a, miR-99b and miR-100 and elevated level of mTOR was also described in prostate cancer and was associated with more advanced tumor stage [52]. Important role of miR-199b-5p was found in medulloblastoma. Garzia et al. reported its lower expression in non-metastatic versus metastatic medulloblastomas and observed that over-expression of miR-199b-5p reduced proliferation of medulloblstoma cells *in vitro* and depleted tumor growth *in vivo*[53].

Although a number of studies reported alterations in miRNAs expression in endometrial cancer only three studies described down-regulation of miR-199b and miR-99 family members [54-56]. Wu et al. compared miRNA expression in ten EEC samples and ten matching endometrial tissues and found decreased expression of miR-99b [54]. Chung et al. reported down-regulation of miR-199b and the fold change of its expression was similar to the one observed in our study [56]. In the study performed by Snowdon et al. the authors compared normal endometrium, atypical hyperplasia (AH) and EEC samples and found that expression of miR-100 and miR-199b was decreased both in AH and in EEC tissues [55]. Moreover down-regulation of those miRNAs was grater in EEC comparing to AH samples [55]. In our study a correlation was found between higher histological grade and lower expression of miR-199b and miR-99a. Although Snowdon at al. were not able to perform correlation analysis between histological grade and clinical stage and miRNA expression due to the small size of studied population, the difference between AH and EEC groups indicated that expression of miR-100 and miR-199b tended to be higher in more advanced stages of endometrial neoplasia [55].

Our study revealed that greater degree of miR-199b and miR-99a down-regulation was attributed to high–grade endometrial tumors. At the same time expression of mTOR kinase mRNA was higher in more histologically advanced tumors, which further supported our hypothesis on the connection between mTOR kinase and studied miRNAs. In addition, there was a significant reverse correlation between expression of all three studied miRNAs and mTOR mRNA expression in matched EEC tissues.

In order to further evaluate the role of miR-99a, miR-100 and miR-199b in EEC we assessed their expression in matched plasma samples obtained from the same group of EEC patients. Surprisingly we found that plasma expression levels of all studied miRNAs were significantly increased in EEC patients in comparison to healthy controls and that expression of miR-99a was higher in patients with clinically more advanced disease. Although the discrepant expression of miR-99a, miR-100 and miR-199b between EEC tissues and plasma is difficult to explain at the time, it could be an additional hallmark of this disease and warrants further investigation. Discrepant expression of another miRNA – miR-92a, was observed in tissue and plasma samples obtained from patients with leukemia, lymphoma and hepatocellular carcinoma [57-59]. In the study performed by Tanaka et al. authors observed that miR-92a, which was attributed oncogenic properties, was highly increased in leukemic cells and down-regulated in the plasma of leukemia patients. Authors hypothesized, that apart from transcribing essential miRNAs, cancer cell might specifically take in exosomes containing miR-92a from the blood, which would result in observed decrease of that miRNA in plasma [57]. Ohyashiki et al. reported similar discordant expression of miR-92a in plasma and tumor tissues in non-Hodgkin’s lymphoma [58]. Shigoka et al. also found that miR-92a was highly up-regulated in tumors and significantly decreased in plasma of hepatocellular carcinoma patients [59].

Another important aim of our study was to evaluate diagnostic and prognostic values of miR-99a, miR-100 and miR-199b expression in tissues and plasma of EEC patients. For this purpose we constructed ROC curves and developed miRNA signatures, which could classify cancer and control samples with high sensitivity and specificity. The 3-miRNA tissue signature developed using multivariate logistic regression was more accurate in discriminating EEC samples in comparison to single miRNAs. Utility of such signature could potentially facilitate diagnosis in poorly differentiated, disseminated and metastatic tumors. Our study also revealed that the 2-miRNA plasma signature (miR-99a/miR-199b) was very accurate in discriminating EEC samples with sensitivity of 88% and specificity of 93%. Survival analysis indicated that both miR-99a and miR-100 were significantly associated with prognosis of overall survival and were independent prognostic factors in a multivariate analysis. One of the great challenges for performing qPCR based miRNA expression analysis in tissues and especially in plasma samples is to use proper normalization strategy, which in most cases bases on reference miRNAs or small RNAs. In the presented study normalization was performed using carefully chosen endogenous controls both for tissue and plasma analysis, which increases validity of our results.

To our best knowledge only two previously published studies attempted to evaluate diagnostic and prognostic values of miRNA expression in endometrial cancer tissues.

Corresponding with our results, Chung et al. reported that expression of miR-199b yielded AUC value of 0.837 in detection of EEC tissue samples [56]. Cohn et al. found that miR-199a was more commonly overexpressed in a subset of patients who did not experience recurrence or death from the disease and its down-regulation in endometrial cancer tissues correlated significantly with shorter progression-free and overall survival [60].

Early diagnosis of endometrial cancer is of immense importance as it facilitates radical surgical treatment and prevents the necessity of adjuvant therapy, which can be connected with severe side effects. However, at present no reliable screening strategy is available for this malignancy. We suggest, that after proper validation tissue and plasma miRNA signatures developed in the current study could have a potential to be applied in a clinical setting. Although plasma miRNA signature was highly accurate in distinguishing between EEC and control samples the number of samples used for its development can limit the value of this finding. Therefore we are planning to further investigate and validate our results in a larger group of patients and in the context of other gynecological pathologies.

## Conclusions

We conclude that increased expression of mTOR kinase in endometrioid endometrial cancer coexists with down-regulation of its targeting miRNAs, miR-99a, miR-100 and miR-199b, which could suggest a new mechanism of mTOR pathway activation in this malignancy. Although functional studies are necessary to directly prove mTOR regulation by studied miRNAs in EEC, presented results along with studies performed in other tumors seem to support this novel mechanism of altered mTOR kinase pathway. In addition, our findings implicate that miR-99a, miR-100 and miR-199b signatures are strongly associated with the diagnosis of endometrioid endometrial carcinoma and after validation in larger groups of patients could be considered promising non-invasive biomarkers for detection and prognosis of this cancer.

## Competing interests

Authors state that there are no potential conflicts of interest.

## Authors' contributions

AT designed the study, collected samples, performed experiments, analyzed data and wrote the manuscript; KT performed experiments, contributed to manuscript, prepared tables and figures; AP and GZ prepared FFPE samples and collected pathological data; MC and PC provided samples, collected and analyzed clinical data; TP and RM gave technical support and conceptual advice. All authors read and approved the final manuscript.

## Pre-publication history

The pre-publication history for this paper can be accessed here:

http://www.biomedcentral.com/1471-2407/12/369/prepub

## Supplementary Material

Additional file 1 **Table S1.** Coefficients, standard errors, odds ratios and confidence intervals of miR-99a/miR-100/miR-199b miRNA signature (backward regression model). Click here for file

Additional file 2 **Table S2.** Overall model fit of the EEC signatures in tissues (miR-99a/100/199b) and plasma (miR-99a/199b). Click here for file

Additional file 3 **Table S3.** Coefficients, standard errors, odds ratios and confidence intervals of miR-99a/miR-199b miRNA signature (backward regression model). Click here for file
