# Supplementary material for: Deregulation of miR-100, miR-99a and miR-199b in tissues and plasma coexists with increased expression of mTOR kinase in endometrioid endometrial carcinoma
Source: BMC Cancer. 2012 Aug 24;12:369. doi: 10.1186/1471-2407-12-369 (PMC3495850; doi:10.1186/1471-2407-12-369)
Supplement: Additional file 1 — Table S1. Coefficients, standard errors, odds ratios and confidence intervals of miR-99a/miR-100/miR-199b miRNA signature (backward regression model). [file 1471-2407-12-369-S1.pdf]

Additional file 1 – Supplementary table 1.

Coefficients, standard errors, odds ratios and confidence intervals of miR-99a/miR-100/miR-199b miRNA signature (backward regression model).

		Coefficient	SE	p	OR	95% CI
b_1	miR-99a	-1.011	0.417	0.015	0.364	0.161 – 0.824
b_2	miR-100	1.047	0.492	0.033	2.849	1.087 – 7.467
b_3	miR-199b	-0.399	0.138	0.004	0.671	0.512 – 0.879
b_0	constant	2.602				
