# Supplementary material for: Deregulation of miR-100, miR-99a and miR-199b in tissues and plasma coexists with increased expression of mTOR kinase in endometrioid endometrial carcinoma
Source: BMC Cancer. 2012 Aug 24;12:369. doi: 10.1186/1471-2407-12-369 (PMC3495850; doi:10.1186/1471-2407-12-369)
Supplement: Additional file 2 — Table S2. Overall model fit of the EEC signatures in tissues (miR-99a/100/199b) and plasma (miR-99a/199b). [file 1471-2407-12-369-S2.pdf]

Additional file 2 – Supplementary table 2.

Coefficients, standard errors, odds ratios and confidence intervals of miR-99a/miR-199b miRNA signature (backward regression model).

		Coefficient	SE	p	OR	95% CI
b_1	miR-99a	2.081	0.874	0.017	8.013	1.444–44.475
b_2	miR-199b	0.865	0.335	0.01	2.376	1.231–4.586
b_0	constant	-4.543				
