# Supplementary material for: Deregulation of miR-100, miR-99a and miR-199b in tissues and plasma coexists with increased expression of mTOR kinase in endometrioid endometrial carcinoma
Source: BMC Cancer. 2012 Aug 24;12:369. doi: 10.1186/1471-2407-12-369 (PMC3495850; doi:10.1186/1471-2407-12-369)
Supplement: Additional file 3 — Table S3. Coefficients, standard errors, odds ratios and confidence intervals of miR-99a/miR-199b miRNA signature (backward regression model). [file 1471-2407-12-369-S3.pdf]

## Additional file 3 – Supplementary table 3.

Overall model fit of the EEC signatures in tissues (miR-99a/ 100/ 199b) and plasma (miR-99a/ 199b).

| miRNA signature | Wald test | | | Hosmer & Lemeshow test | | |
|---|---|---|---|---|---|---|
| | $\chi^2$ | df | p | $\chi^2$ | df | p |
| miR-99a/ 100/ 199b | 18.22 | 3 | 0.0004 | 11.11 | 8 | 0.195 |
| miR-99a/ 199b | 22.8 | 2 | <0.0001 | 5.245 | 8 | 0.731 |
